# Role of Mass Media and Public Health Communications in the COVID-19 Pandemic

**DOI:** 10.7759/cureus.10453

**Published:** 2020-09-14

**Authors:** Ayesha Anwar, Meryem Malik, Vaneeza Raees, Anjum Anwar

**Affiliations:** 1 Internal Medicine, Allama Iqbal Medical College/Jinnah Hospital, Lahore, PAK; 2 Biotechnology, Harvard University, Cambridge, USA; 3 Psychiatry, Fatima Jinnah Medical University/Sir Ganga Ram Hospital, Lahore, PAK; 4 Medicine, University of Washington, Seattle, USA; 5 Anesthesia, University of Washington School of Medicine, Seattle, USA

**Keywords:** public health and safety, public healthcare, covid-19, effects of social media, infodemic, telemedicine, mass media, healthcare literacy, healthcare policies, communication in healthcare

## Abstract

In Dec 2019, a novel pathogen emerged, and within weeks, led to the emergence of the biggest global health crises seen to date. The virus called ‘SARS-CoV-2’, causes coronavirus disease which was named ‘COVID-19’ by the World Health Organization (WHO). The speedy spread of this infection globally became a source of public worry and several unknowns regarding this new pathogen created a state of panic. Mass media became the major source of information about the novel coronavirus. Much like the previous pandemics of SARS (2003), H1N1 (2009), and MERS (2012), the media significantly contributed to the COVID-19 infodemics. In this review, we analyze the role of mass media and public health communications from December 31, 2019 to July 15, 2020, and make scientific inferences. The COVID-19 pandemic highlights multiple social, cultural, and economic issues arising from the media’s arguable role. The racial prejudices linked to the origin of the virus prevented collaborations among scientists to find a solution. Media coverage of coronavirus news during geographical lockdowns, extended quarantines, and financial and social hardships induced fear and caused psychological stress. Domestic and elderly abuse upsurged. The unscientific cures and unverified medicines endorsed by the politicians and fake doctors proved harmful. The media played a worldwide role in coronavirus disease tracking and updates through live updates dashboard. The media allowed for timely interventions by the Center For Disease Control And Prevention (CDC) and the World Health Organization (WHO), enabling a rapid and widespread reach of public health communications. We saw an upward trend for the promotion of health and hygiene practices worldwide by adaption of safe health practices such as increased hand washing, use of face coverings, and social distancing. Media reinforced illness-preventing guidelines daily, and people were encouraged to use telehealth to meet their healthcare needs. Mass media has an imperative role in today’s world and it can provide a unified platform for all public health communications, comprehensive healthcare education guidelines, and robust social distancing strategies while still maintaining social connections. It can enable equal access to healthcare, end discrimination, and social stigmatization. The role of media and public health communications must be understood and explored further as they will be an essential tool for combating COVID-19 and future outbreaks.

## Introduction and background

COVID-19 is a global infectious disease that emerged from Wuhan in the Hubei province of China in December 2019. It has spread to 210 countries throughout the world. World Health Organization (WHO) declared it a pandemic on Jan 30, 2020, and raised international public health concerns for it [1]. As of Jul 15, 2020, more than 13 million people have been affected by this disease. To date, around 0.6 million deaths have been reported. It has proved far more fatal than other coronavirus family members, with a fatality ratio of 1.4% (varying slightly among countries) [2]. Between Dec 31, 2019, when the Chinese authorities declared their first case of pneumonia of unknown etiology to until Jan 3, 2020, a total of 44 cases were reported to WHO. However, the causal agent was not identified during this reported period. Subsequently, the novel coronavirus was identified on Jan 7, 2020, and its genomic sequence was shared with the world [3]. WHO named the disease as ‘COVID-19’ and causative virus as ‘SARS-CoV-2’ on Feb 11, 2020 [4]. It has been named due to its genetic resemblance to the coronavirus that caused the SARS outbreak of 2003. The other members of the family include SARS coronavirus SAR-CoV and MERS coronavirus MERS-CoV.

The disease primarily affects the respiratory system with symptoms ranging from fever, cough, and mild shortness of breath, to severe desaturation, causing respiratory failure. Despite the lung damage in the form of adult respiratory distress syndrome (ARDS), there are reports of the novel virus creating a thromboembolic condition in the body and hence causing myocardial infarction and pulmonary embolism. It can also result in kidney failure in several patients. Modes of spread range from droplets, airborne, or feco-oral to contact spread. There is news of viruses staying viable on surfaces from a few hours to many days. This heterogeneous spectrum of disease is concerning and one of the reasons for the increased fatality of the disease. 

These aspects create a public worry and force the general public to seek help from the most accessible ways available to them. For most people, it’s either the internet or media, which includes print, as well as broadcast options. The internet is considered a worldwide media. In an epidemic or pandemic, controlling the spread of disease is a basic requirement. It requires early recognition of symptomatology, prompt diagnostic measures, effective home and hospice management, and appropriate preventive steps. This in turn, requires the role of varying departments ranging from government to healthcare, to the media, to the general public itself. Whenever a new virus or bacterial disease emerges, it goes through localized transmission, amplification in the spread, and finally, the remission with successful measures. The controlling measures are taken at each step of the chain. They include anticipation about the likely widespread infection, early detection, effective containment, control and mitigation measures, and lastly, eradication. According to WHO, it involves the coordination of responders, proper health information system, and managing communication risks. Media plays a crucial role at each step. The method of news reporting modifies the behavior of people and their attitudes. This was studied in the H1N1 influenza epidemic in the Shaanxi province of China in 2009. In a study published in 2016, Yan Q. et al. showed how people’s response can change with media reports and, hence, can affect emerging disease control [5]. Media reports of the disease spread during the H1N1 pandemic in 2009 raised fear and awareness among people. On the one hand, it helped people to adopt essential protective measures. On the other hand, some people started stigmatizing diseased persons from inappropriate articles published in some newspapers [6]. This is an example that indicates that the interaction between media awareness and disease control is a two-directional approach.

To study the media impact and disease dynamics, the media impact model was designed after the pandemic of SARS 2003-2004. This was inconclusive of the overall positive or negative impact of media, thus prompting the need to expand the model and studying its effects [7]. In the MERS outbreak in 2012, again, the media played its role. With advancements in technology and an increase in the accessibility of the internet to the common man, public awareness increased manifold, thus urging better adherence to essential public health measures. The role of social media in the MERS epidemic in 2012 and the H7N9 epidemic in China was studied, showing a stronger reaction in the H7N9 epidemic. It further delineates the importance of the topic of discussion. This article will examine the role of the media in this COVID-19 pandemic and its impact on the general public.

## Review

Role of media in COVID-19

Origin

In December 2019, the reports of patients with viral pneumonia in China surfaced. The origin was related to the Huanan Seafood’s whole market. Researchers established that the disease has most likely originated from bats, mutated to infect humans, and transmitted by droplet routes among humans. Since the origin occurred in a wet market, the media criticized the Chinese for consuming live animals like bats, snakes, and dogs. Previously known as “Wuhan virus” and “China coronavirus,” it was subsequently called 2019-nCoV, and then finally, on February 11, the World Health Organization (WHO) gave the disease an official name, "Covid-19". In spite of this, renowned world leaders ignorantly kept calling it “Chinese virus” which left concerning effects in international communities [8,9]. Asian people were stigmatized and called by the name ‘Corona’ on the streets. Many reported incidents of such racial slurs which exaggerated already existing prejudices among people. This happened in SARS 2003 in Toronto which caused the xenophobic reaction, and similarly, was repeated with the coronavirus pandemic. It had a significant impact, leading the Chinese government to order the closure of all farmer’s markets and put a ban on eating live animals. This was an essential step in halting the spread of the virus. However, it also created a false sense of security among the rest of the world, and no one took the necessary precautions when the cases were limited to China.

Another news was that the ongoing bioweapons research in Wuhan universities has led to the emergence of the virus. It resulted in tweets about USA army troops bringing the virus to Wuhan while attending military games in Oct 2019. The use of media to spread rumors has been happening for a long time. For example, in 1985, the CIA was blamed for manufacturing the AIDS virus, prompting similar reactions in the public’s perception of the spread and handling of the virus. Such conspiracies create differences among nations, form unseen boundaries, and hinder the collaboration of scientists throughout the world in finding solutions. This recurred in the development of the COVID-19 vaccine as well, and the task became a competition for innovation and technology between Washington and China, rather than a solution for public well being. Thus, the goal of the world’s two largest economies became to win the battle of supremacy and achieve ultimate power [10].

Emotional and psychosocial aspects

Social media usage has increased manifold and thus, has a number of available platforms, including Facebook, Twitter, YouTube, Instagram, Snapchat, WhatsApp, and Reddit, along with their Chinese equivalents WeChat, Weibo, Tencent, Tik Tok, and Toutiao. People have become accustomed to posting every aspect of their lives on social media. This includes their achievements, worries, and travels on a daily and hourly basis. Since the lockdown, there has been an 87% increase in social media usage by the people [11]. People started gathering information posted on the groups and unknown pages and believing them. Religious pages also started attracting people amid crises by spreading unscientific information regarding the prevention and treatment of the virus.

The implausible claim of the virus affecting the geriatric population made the young vulnerable. This ultimately resulted in a large number of young affected by the virus. This unscientific and unproven fact spread like wildfire in the media and made the elderly go through many psychological and physical terrors. There were reports of old people being removed from family homes, and increased emotional abuse cases among them. This changed the public’s perception of the elderly population and caused increased depression among them based on society’s prompt reactions. In contrast, the millennials, college students, and high schoolers made their way to beaches to party in anticipation of their annual Spring Break Weekend, which later proved hazardous. Social media spread this spurious information regarding the virus that played with the minds of people who started refuting the importance of social distancing. Moreover, disruption in professional lives and sticking to social media in all this free time, highlighted the problems like racism and wealth inequality. Many cases of domestic violence were also reported. This further adds to the already growing depression due to quarantine.

Stress is the normal physiological response of human beings to variable unfavorable situations occurring in life. Those who are unable to control it go through anxiety or phases of depression. Depression can present itself in physical and psychological forms, which vary from person to person. To overcome it, some behavioral changes or medications may be required. The critical role of the media is to keep people connected, well informed, and entertained. The positive impact of the media was shown in the COVID-19 crises in promoting emotional stability among people. Pages and groups on platforms like Facebook and Instagram started posting videos regarding physical and mental health. Many relaxation exercises were advertised, and books were made accessible free of charge. Scholastic ensured free and feasible book attainability for young kids [12]. There were many other similar examples. Many people related to institutions started free online educational activities for kids of various ages. Numerous groups were formed, encouraging people in homeschooling with daily postage of worksheets for kids. Activities for toddlers helped guardians at home to a level that they stayed assimilated in healthy exercises daily. Moreover, stores also started selling educational toys on huge discounts with widespread advertisements through the media.

The CDC's many beneficial guidelines for preventing COVID-19 were reinforced among people through prominent advertisements on commonly used social media platforms. Facebook, Instagram, and television media posted the importance of ‘social distancing’ and ‘stay at home’ through free of cost and frequent, widespread ads. The printed media was utilized by supermarkets to promote their stores following the social distancing protocols. During road and air travel, there is continuous mention of ads like ‘Stay home, stay safe,’ ‘Face covers mandatory in public,’ ‘COVID-19: less is more, avoid gatherings’, ‘give extra space with each other and on the road,’ and ‘wash your hands, stay healthy, avoid COVID-19’. This repetition is essential to consolidate the role of them in preventing the disease spread. This campaign was run extraordinarily by the media using all resources and its subtypes. 

Telemedicine

Telemedicine is a service provided remotely to patients for health-related advice, solving queries, and monitoring diseases via a secure connection, thus maintaining patient-doctor confidentiality. However, historically, the telemedicine service has remained under-utilized. In 2017, a survey study in the USA showed that 82% of people do not use this service [13]. The underlying cause may be related to limited availability, especially in rural areas of the world or cultural reasons. However, the government has always employed this in cases of tornadoes or disaster management. It uses a wide array of technologies like audio-video sessions, telephonic discussions, and integrated clinical information systems to help deal with problems faced by sick people in the community [14]. In the COVID-19 era, telemedicine has become the backbone of clinical practice. Virtual treatment of patients started at the beginning of the pandemic. People became scared of going to hospitals even for major problems. A helpline was also set up, enabling people to decide whether their symptoms accounted for COVID-19 testing. Media was used to promote it and hence, maintain norms of the lockdown. 

No checks were maintained initially, and many “fake doctors” started using prominent social media platforms for this purpose. This specifically did the damage of spreading erroneous information regarding the virus and additionally persuaded beliefs in simple enigmatic treatments, thus causing people to become careless and assist the spread of the disease. For instance, rumors were spread about the use of humidification and steam for thwarting the disease and a video was aired on Facebook live lasting 40 minutes explaining the use of ‘steam inhalation’ as a way of killing the virus. In the video, a ‘technician’ elucidated the usage of boiled water mixed with sea salt and citrus peels for 15 minutes which was viewed 2.4 million times [15]. This provides only symptomatic relief during a common cold, and can seriously damage eyes, face, and airways through the heated water vapors.

Steam inhalation was just the beginning; many other local treatments appeared on social media and spread swiftly via messages. COVID-19 has no cure yet, and consequently, any potential cure has been shared without genuine medical research. It was widely emphasized to take vitamin C to boost immunity and strengthen the body as a preventative measure against the disease. Vitamin C is highly effective in combating the common cold. A meta-analysis showed the role of prophylactic intake of vitamin C in reducing the duration of disease but no effect on incidence and severity [16]. Vitamin C only improves resistance against pathogens by enhancing the immune system. The precise role of Vitamin C is not known, which can incite indiscreet practices in individuals to accept that it is more potent than it is. A clinical trial is being conducted at Cleveland Clinic to study the role of vitamin C as a supplementary medication for shortening the duration of COVID-19 [17].

In this time of psychological crises, various mental health organizations have developed a comprehensive approach for managing the rapidly rising mental disease load. Loneliness due to social isolation, anxiety about disease uncertainty among affected, financial losses due to business closures, despair due to discrimination, and insomnia from boredom are some of the psychological problems faced by people. There has been an increase in the number of suicides. Domestic violence is on the rise. Also, grief and bereavement of losing known people are being recognized. An expert team has been formed to serve in this situation. Online mental health services are working to combat mental diseases, and psychiatrists and psychologists on the internet provide free consultations. Social media is contributing to mental health education for the public.

SARS-COV-2 tracking tools

Amidst all the chaos and panic created by the virus, news channels played no less of a role in spreading panic. Different news channels started giving inconsistent information regarding the virus, number of cases and deaths worldwide. At this time of trouble, the map created by the John Hopkins Center of Systems Science and Engineering came as a shining light. It established a record-based data of affected people and deaths. Professor Lauren Gardner and her graduate student built the dashboard, which provided a tool for public health authorities, researchers, and the general public to track the reported cases and deaths in a friendly manner [18]. This was posted online on the internet for public use on Jan 22, 2020. Mapping the virus spread across the world helped with the uncertainty about its geographic spread and therefore, facilitated governments of various countries to adopt and begin timely actions.

This shows how the internet can play a positive role in crises and what it did in COVID-19. It paved the way for others to form tracking systems. Worldometer is another example, giving us live statistics and updated news regarding coronavirus [2]. It paved the way for others to form tracking systems and enable a common person to maintain their own excel data and use it for research or knowledge. Here is an example:

**Figure 1:**
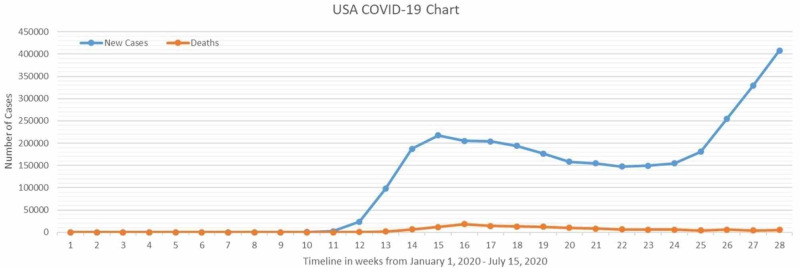
Graphical representation of new COVID-19 cases and deaths per week from the first to 28th week of 2020, (Jan 1, 2020 - Jul 15, 2020). Data taken from the Worldometer.

WHO has unveiled its Arc Geographic Information System (ArcGIS) Operations dashboard for COVID-19 on Jan 26, 2020, which maps and lists the number of cases and deaths [19]. It shows an epidemic curve that represents the number of cases by date of reporting. It also has a section that provides links to additional authentic information regarding COVID-19. The main goal is to map the worldwide spread of misinformation and confusion.

This breakthrough of media helped the countries predict how this outbreak is going to unfold and, hence, enabled them to put up necessary measures at appropriate times. This also made it possible for scientists and researchers to develop different prediction models regarding the pandemic’s course by formulating and guiding about the peak of cases and deaths at each place. Additionally, it provided hope in this grave situation by showing the number of recoveries. Now, computer-based spatial analyses integrating physio-epidemiological methods for identifying new likely outbreak centers are also being done.

Publicity of Chloroquine 

Media is the primary source of information and plays a vital role in educating the masses. However, when overly eager sources spread information without proper verification, not only can it be harmful but it can have unintended consequences. The hydroxychloroquine (HCQ) example fits the scenario. HCQ, a lyso-somatotrophic agent, is an approved drug to treat malaria and some autoimmune diseases. Its propensity to fight certain viruses is explained by its role in blocking the function of lysosomes. It is postulated that at an acidic pH, certain viruses, after being internalized through the plasma membrane of cells, can fuse with lysosomal membranes, thus entering the cells and replicating. Chloroquine being a weak base, enters into lysosomes and raises the pH of the lysosome. As the pH rises, lysosomal enzymes fail to function, and viruses requiring acidic pH can no longer enter the cells. The productive role of chloroquine against SARS-CoV- 2 has been demonstrated in vitro [20]. The efficacy of HCQ in humans is yet to be determined. Several deaths were reported from chloroquine misuse, following Trump's endorsement of this drug as a "game changer" [21]. The first trial was done by Chinese investigators, followed by the French, which showed the drug’s efficacy in the duration of symptoms, radiological picture, and hospital stay. These trials were limited, non-randomized, and done on a small scale. Based on these limited trials, ICMR (Indian Council of Medical Research) recommended using chloroquine in healthcare workers and asymptomatic contacts without significant data. However, the CDC's clinical guidance on the use of chloroquine for prophylaxis was lacking because of the absence of results from Randomized Control Trials (RCTs).

**Figure 2:**
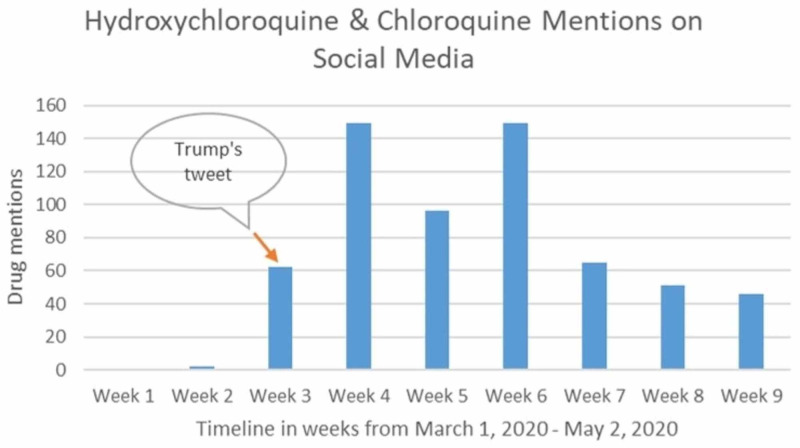
A Graphical representation of the social media mentions of hydroxychloroquine and chloroquine. We used the tool Awario.com to generate the drug mentions using keywords, Hydroxychloroquine and Chloroquine, and created a bar graph showing the social media mentions of the two drugs before and immediately after President Trump’s tweet on March 19, 2020.

Currently, several big pharmaceutical companies are pursuing the treatment and prevention of the novel coronavirus. However, aside from remdesivir created by Genentech, we do not have a promising drug for COVID-19 treatment. Thus, when chloroquine appeared as a hope, it sparked the interest of the media as a potential cure. Some politicians created hype and promoted chloroquine by declaring it effective against the novel coronavirus and referred to it as the ‘biggest game-changer in the history of the world (Figure 2) [22].

This misstep or honest mistake proved hazardous for the public. Hydroxycloroquine is inexpensive, easily available in malaria-endemic regions, and became highly sought after following its publicity on several media platforms. It disappeared from the market like a ghost, prices skyrocketed, and vendors started stocking up on it. This created a supply chain deficit and its shortage left a profound impact on people using it as a management for their autoimmune diseases. Healthcare providers noted increased number of lupus disease flare ups and a spike in rheumatoid arthritis cases.

Moreover, many deaths were reported because of it's overuse. Chloroquine can have many adverse effects, the vast majority of which represent electrical arrhythmias in the heart like ventricular tachycardia, long QT syndrome, torsades de pointes, and sudden death. Also, without the results of studies, a safe dosage in COVID-19 was unknown. In Nigeria, at least two people were reported to have overdosed from the drug, and a man in Arizona died after taking it as self-medication, while his wife got critically ill [23, 24]. Chloroquine gained all this attention after a small non-randomized clinical trial of just 36 patients from France [25]. However, a subsequent randomized clinical trial using high dose chloroquine was halted in phase 2b due to the lethal effects of the drug observed [26]. The FDA also had to issue a warning against chloroquine for COVID-19 after the results of the study were shared [27].

In just a small period, chloroquine was portrayed over social media as a wonder drug with a coronavirus cure, misleading the public about its effectiveness and rendering negative consequences. This shows that politicians should not be allowed to provide scientific information to the general public, especially using the media as a platform.

Discussion

The COVID-19 outbreak spread worldwide within days due to the rapid advancements in technology, which has turned the whole world into a global village. Global traveling can transform epidemics to pandemics within the blink of an eye. In the present era, the media has become a powerful resource that affects and controls the disease outcomes in several ways. Owing to the vastness and variability in different media types, it can play both positive or negative roles. In COVID-19, the media has made a unique mixture of varied contributions. Here are the few things that the media should do in any outbreak to play its role effectively and practically:

Our proposed mass media model has six major components and we recommend evaluating the role of media according to how it performs in each category. This evaluation can serve as valuable feedback for timely and effective interventions using mass media as a tool to prevent and control all future outbreaks.

Public health communication

Whenever there is an outbreak, people tend to turn to the media for information. In COVID-19, Sprinklr recorded a count of nearly 20 million mentions of coronavirus on March 11. Extensive varying news coverage is enough to make the public feel dazed. Additionally, the spread of unscientific news by instant message technology creates a panic. This is also known as ‘headline stress disorder.’ In this pandemic, false and misleading information spread unchecked by using different types of media. Social media groups realizing this started utilizing third-party fact-checkers to limit the dissemination of the concocted knowledge. This happened late, and the damage was already done. It should be noted that:

-Only information that pertains to WHO or CDC guidelines should be allowed to be posted on social groups or aired on televisions.

-Each media source should have a way of connecting people to credible sources by having special tabs or pop-ups.

-Media should act as a bridge for people in need to reach health officials and the local government for their problems.

-Banning advertisements for medical equipment and drugs which have not been proven to have a definite role.

-Provide a platform to people to seek financial or social help from the people of the community.

-Scientific and political statements should be kept separate and properly categorized.

-Encouraging and appreciating the workforces trying to combat the disease.

Health education

The pivotal roles that the media can play in the current pandemic is promoting physical and psychological health measures and ensuring resilience in people belonging to different age groups and socioeconomic conditions. In COVID-19, it is a prerequisite to ensure public knowledge regarding the following things:

-Social distancing of 6 feet (2m) and its importance.

-Appropriate usage of face masks: One way to encourage the use of facemasks among the masses is for the government to provide them rather than people having to pay out of pocket to purchase them.

-Proper guidelines for recognizing, diagnosing, and managing the disease: Online symptom checkers can create unnecessary panic. 

-A comprehensive set of guidelines can be made available throughout the internet from a single source like CDC or WHO, who do their due diligence before releasing them. Steps should then be taken to mandate the use of these guidelines worldwide. 

-Mental health should be prioritized along with physical health. 

-Experts should be allowed to participate only after verification of valid licenses and degrees online.

-During this pandemic, COVID-19-specific mobile apps can be developed that keep users updated about the pharmacies and their inventory, grocery stores and their inventory, hospital beds availability, Emergency Room (ER) wait times, and urgent care clinics in their localities.

Strategies for social distancing

Social isolation is associated with psychological changes among individuals. It incorporates feelings of loneliness, anxiety, and depression. It can be propagated and exacerbated by the conflicting role of the media. Media, however, can prevent this by adopting certain strategies like:

-Mental health programs to eradicate depression and anxiety for both the general public and health workers.

-Videos for relaxation exercises to keep bodies and minds healthy and fit.

-Education services provided to children of each age group.

-Social media groups to keep positive energy running among people and ensure adherence to public health measures.

-Connecting families and friends across the world by video chats, texts, and phone calls in times of social isolation.

Reduction of stigma, discrimination and prejudice

Media became the platform for spreading prejudice among people by spreading stories about the origin of the virus. Racial and socioeconomic discrimination became apparent during the quarantine. Inaccessibility to equal healthcare made the situation worse. Under such unprecedented circumstances, the media has the potential to unite people and end discrimination by spreading awareness. It can also be an excellent resource for information verification. However, this can only be possible through the responsible use of media where proper checks and balances are in place. This way, it can help prevent the spread of rumors and end stigmatization of those affected by COVID-19.

Telemedicine

Telemedicine has been a chief strategic step in all unprecedented times. It was employed in disasters, tornadoes, and wars. In the COVID-19 pandemic, it has been a useful tool not only for those needing virus testing but also for dealing with people facing other health problems. There is a need to implement specific essential measures from the start of any pandemic. These include telephone helplines and online doctor services, pharmacy delivery of drugs at the doorstep, and managing diseases by video calls.

Managing infodemics

"Infodemic" stems from two words, "information" and "epidemic", and refers to a rapid and far-reaching spread of both accurate and inaccurate information about a disease. During a pandemic, when facts are often mixed with rumors, it becomes difficult to learn essential information about an issue. Infodemics, like epidemics, can be managed. WHO states in its publication “Managing epidemics: Key facts about major deadly diseases” (May 2018) that there are three main aspects to ‘outbreak risk communication,’ that must be worked out:

-Experts and concerned authorities should rapidly employ ways to relay necessary and concerning information to the public as soon as possible using mass media, including print media (brochures, pamphlets, newspapers), television, internet, and social media. Translational communication methods should be used to help populations as a whole and hence, remove social and cultural biases.

-Authorities need to address the fears, concerns, perceptions, and anxiety of people and devise ways to answer each query of each individual regarding anything. Again, the media can be utilized for this. Health surveys can be conducted via social media, television, or radio to tailor this. Views of people affected and interventions done for them can be wired via messages and managed.

-Controlling rumors and misinformation is required. Social media, which is a source of such propagation, can take fundamental measures to reduce them in a timely way. There should be ways devised to listen and correct the misinformation.

## Conclusions

COVID-19 is a global crisis that has spread throughout the world at a dangerously fast pace. Mass media plays a huge role in circulating information, influences the public behavior and can curtail the spread of disease. In this article, we discussed the positive and negative impacts of media and proposed steps that can be taken to use media effectively in outbreaks. Furthermore, we recommend creating a model to evaluate the media response at the end of each epidemic and pandemic. This evaluation can serve as a feedback for the media to help devise better and more effective strategies to control and prevent subsequent outbreaks.
